# Glucose-6-Phosphate Dehydrogenase Deficiency and Sickle Cell Trait among Prospective Blood Donors: A Cross-Sectional Study in Berekum, Ghana

**DOI:** 10.1155/2016/7302912

**Published:** 2016-09-14

**Authors:** Patrick Adu, David Larbi Simpong, Godfred Takyi, Richard K. D. Ephraim

**Affiliations:** ^1^Department of Medical Laboratory Technology, School of Allied Health Sciences, University of Cape Coast, Cape Coast, Ghana; ^2^Holy Family Hospital, Berekum, Brong-Ahafo Region, Ghana

## Abstract

*Background.* Blood transfusion is a therapeutic procedure usually undertaken in patients with severe anaemia. In Ghana, severe anaemia is mostly due to malaria caused by severe* Plasmodium falciparum* infection, road traffic accidents, and haemoglobinopathy-induced acute haemolysis.* Method.* This cross-sectional study evaluated coinheritance of sickle cell haemoglobin variant and G6PD enzymopathy among individuals that donated blood at the Holy Trinity Hospital, Berekum, in the Brong-Ahafo Region, Ghana. Demographic data and other pertinent information were captured using questionnaire. Sickle cell haemoglobin variants were determined using cellulose acetate electrophoresis (pH 8.6). Qualitative G6PD status and quantitative G6PD enzyme activity were determined using methaemoglobin reduction and Trinity Biotech G6PD test kit, respectively.* Results.* Prevalence of sickle cell trait (SCT) and G6PD enzymopathy coinheritance was 7%. In addition, 19.5% of the donors had 10%–60% of normal G6PD enzyme activity suggesting that these donor units are prone to stressor-induced acute haemolysis when given to recipients. Mild G6PD activity (*p* = 0.03, OR: 2.410 (CI: 1.049–5.534)), commercial (*p* = 0.020, OR: 5.609 (CI: 1.309–24.035)), and voluntary (*p* = 0.034, OR: 2.404 (CI: 1.071–5.397)) donors were significantly associated with SCT.* Conclusion.* Screening for red cell pathologies must be incorporated into existing protocols for populations with high incidence of haemoglobinopathies to protect high-risk recipients.

## 1. Introduction

Blood transfusion is a therapeutic procedure usually undertaken in patients with severe anaemia. In Ghana, severe anaemia is mostly due to malaria caused by severe* Plasmodium falciparum* infection, road traffic accidents, and haemolytic episodes in individuals with haemoglobinopathies such as sickle cell anaemia and/or thalassaemia [1, 2]. It is estimated that most of the transfusions are undertaken in children under 5 years in response to severe* falciparum* infection [1]. In addition to the transfusion, such children may also be receiving antimalarial drugs (e.g., quinine and primaquine) with potential to cause oxidant stress. In these individuals, the optimal survival of the transfused red cells is of paramount importance so as to prevent adverse transfusion outcomes [3]. To ensure the safety of blood and blood products for prospective recipients, screening protocols for transfusion-transmitted infections such as human immunodeficiency virus (HIV), hepatitis B and C, and syphilis are mandatory [4]. It has been postulated that selective pressure caused by the endemicity of* Plasmodium falciparum* infection in Ghana, and Sub-Saharan Africa in general, has led to high prevalence of certain haemoglobin variants (e.g., HbS) and/or red cell enzymopathies (e.g., G6PD deficiency) as these have been shown to offer survival advantages [5–7]. The high prevalence of these inherited red cell pathologies suggest that measures should be taken prior to donation and subsequent transfusion of such units to other individuals who might have also inherited these red cell pathologies. Some have argued that donor blood from those heterozygous for haemoglobin S or haemoglobin C should not be used for either exchange transfusion or neonatal transfusion [8, 9]. Others have also argued that those who have had previous oxidant stress-induced haemolysis as a result of G6PD enzyme deficiency must be permanently deferred for the safety of both the donor and prospective recipient [8]. Thus, knowledge of the red cell pathologies inherent in a given donor unit may be important for ensuring maximal benefit to the potential recipient.

Howes et al. estimated that the prevalence of G6PD deficiency could be as high as 32.5% across sub-Saharan Africa [10]. The WHO also estimates the prevalence of G6PD deficiency in Ghana to be 15–26% [11]. Moreover, the prevalence of sickle cell trait (SCT) in Ghana has been estimated to be between 20% and 40% [12]. However, previous studies carried out in Ghana and the subregion did not measure the G6PD enzyme activity in the donors [13, 14] and could not therefore categorize the classes of the G6PD enzymopathy in the studied donors as per the WHO recommendations [11]. In addition, the existing predonation screening protocols in Ghana do not assess for either red cell enzymopathies or haemoglobinopathies in spite of the high prevalence of these pathologies. In this study, we sought to screen for haemoglobin variants and G6PD status/enzyme activity in donor blood that had been declared fit for transfusion as per the existing predonation screening protocols. The aim was to assess whether recipients with certain medical conditions such as severe falciparum malaria or vulnerable groups like neonates requiring exchange transfusions of blood products are adequately protected from products that have the potential to complicate clinical outcomes.

## 2. Materials and Methods

### 2.1. Study Site/Study Design

**Figure 1:**
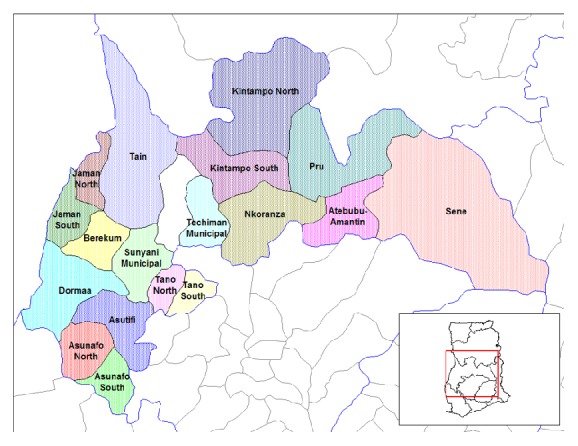
Map of Brong-Ahafo Region of Ghana (source: Google map).

This was a hospital based cross-sectional study carried out from August 2015 to January 2016 at the Holy Family Hospital at Berekum in the Brong-Ahafo Region (Figure 1). Holy Family Hospital (HFH) is a Catholic health institution which serves as the Municipal Hospital for Berekum with Level C rating, for medical care in the PHC strategy.

### 2.2. Study Population

All blood donors ≥18 years who tested negative for all the transfusion-transmitted disease screening assays were enrolled. A total of 200 donor samples were collected for the study using a convenience sampling technique. All samples were obtained from donor blood collected in citrate phosphate dextrose adenine 1 (CPDA-1) and assayed within 24 hours of collection. Questionnaires were used to capture demographic data, medication history, donor type and history, and other pertinent information. All prospective donors on medications (Such as cotrimoxazole, aspirin, fansidar, and nitrofurantoin.) known to affect G6PD enzyme activity were excluded [15].

### 2.3. Ethical Considerations

Experimental protocols were approved by the institutional review board of University of Cape Coast and Holy Trinity Hospital ethical review board (UCCIRB/CHAS/2015/61). The qualitative and quantitative G6PD assays were performed independently and were blinded to remove potential operator bias.

### 2.4. Qualitative G6PD Assay

The methaemoglobin reduction test assay previously described [1] was used to qualitatively assay for G6PD status. For each sample, three tubes were set as test (T), normal (N), and deficient (D) as internal quality controls to validate the results.

### 2.5. Quantitative G6PD Assay

Quantitative G6PD activity was measured in duplicate per sample using the quantitative G6PD kit from Trinity Biotech (catalog number 345-B; Trinity Biotech PLC, Bray, Ireland) according to the manufacturer's instructions. As per the manufacturer specifications, normal, intermediate, and deficient Trinity controls (catalog numbers G6888, G5029, and G5888, resp.) were run using the same method on each day of testing. 10 *μ*L whole blood collected in CPDA-1 was added to 1 mL G6PD reagent solution and incubated at room temperature for 5 minutes. Two milliliters (2 mL) of substrate was added to the solution and mixed by inversion. The initial absorbance and final absorbance of all samples were measured using Junior Selectra chemical analyser and G6PD enzyme activity calculated in accordance with manufacturer's protocol. G6PD activity values were expressed in units per gram haemoglobin (Hb). Haemoglobin concentration was determined using Sysmex-XS haematology analyser (Sysmex Corporation, USA).

### 2.6. Haemoglobin Electrophoresis

The cellulose acetate method of electrophoresis was employed to determine the haemoglobin variants of all blood samples in accordance with protocols previously described [16]. Each sample was washed four times in physiological saline and subsequently lysed in carbon tetrachloride (CCL4). The lysates were applied to the cellulose acetate paper and run for 30 minutes at 250 V and current 50 mA. For each electrophoretic run, combination of hemolysate from a sickle cell trait (AS) and HbC trait samples (ASC) served as the control.

### 2.7. Data Analysis

Data were analysed using GraphPad prism 5.01 for Windows (GraphPad Software Inc., USA). Data were analysed for normality using D'Agostino and Pearson omnibus normality test and appropriate test selected for parametric and nonparametric data accordingly. For nonparametric data, multiple comparisons were undertaken using Kruskal-Wallis test with Dunn's posttest to determine statistical significant differences between groups. However, logistic regression analyses were undertaken using IBM SPSS version 16 (IBM Corporation, USA).

## 3. Results

As demonstrated by cellulose acetate electrophoretic mobility assay, 68% of the donors had neither the sickling haemoglobin (HbS) variant nor any qualitative G6PD defect. However, whereas 7% of the donors had both full qualitative red cell G6PD enzyme defect and haemoglobin AS phenotype, 12.5% of the donors had haemoglobin phenotype AS only, while 10.5% of the donors had full qualitative G6PD enzyme defect only (Table 1).

Whereas none of the donors had any knowledge about their G6PD status, only 3% of the study participants knew about their sickle cell haemoglobin status. However, 24% and 37% of the donors who had no knowledge about their G6PD and sickling haemoglobin status, respectively, had had previous blood donations. Overall, 19.5% of the blood donors had either HbS variant or G6PD enzymopathy (Table 2).

Of the 200 donors, 4% were commercial donors, that is, those donating for financial rewards (50% of which had HbAS phenotype), 76% were replacement (i.e., donation to a relative) donors (15.8% of which had HbAS phenotype), and 20% were voluntary blood donors (30% of which had HbAS phenotype) (Table 3).

50.5% of the study participants belonged to the 18–29 age group, whereas 40.5%, 8.5%, and 0.5%, respectively, belonged to the 30–39, 40–49, and 50–59 age groups, respectively (Table 4).

**Figure 2:**
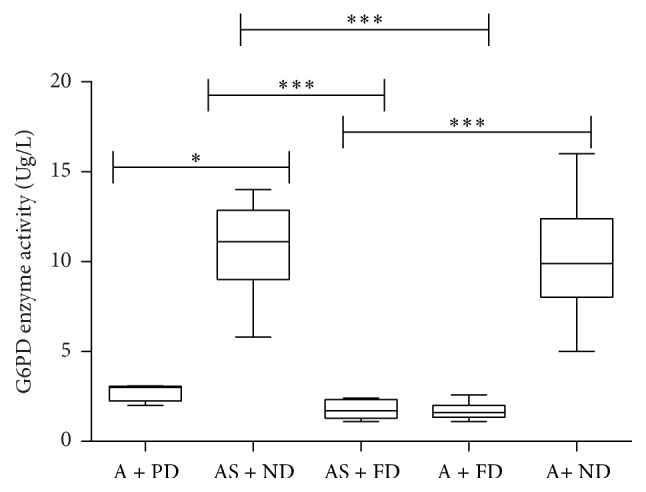
Comparison of red blood cell G6PD enzyme activity levels of blood donors. G6PD activities of red cells were measured using quantitative G6PD kit from Trinity Biotech and calculated against the donor haemoglobin levels. Statistical differences between G6PD enzyme activities were estimated using Kruskal-Wallis test with Dunn's posttest for multiple comparisons. A: haemoglobin A; S: haemoglobin S; ND: no qualitative G6PD activity; FD: full qualitative G6PD defect; PD: partial qualitative G6PD defect (^*∗*^ *p* < 0.05; ^*∗∗∗*^ *p* < 0.0001).

The red blood cell G6PD enzyme activities were significantly reduced in donors who demonstrated either partial or full qualitative defect when compared to donors with no qualitative enzyme defect (Figure 1; *p* < 0.05 (A + PD versus AS + ND); *p* < 0.001 (AS + ND versus AS + FD; AS + ND versus A + FD; AS + FD versus A + ND); A: haemoglobin A; S: haemoglobin S; ND: no qualitative G6PD activity; FD: full qualitative G6PD defect; PD: partial qualitative G6PD defect). However, this reduced G6PD enzyme activity was independent of the haemoglobin phenotype of the donor (Figure 2; *p* = ns (AS + ND versus A + ND; AS + FD versus A + FD)).

**Figure 3:**
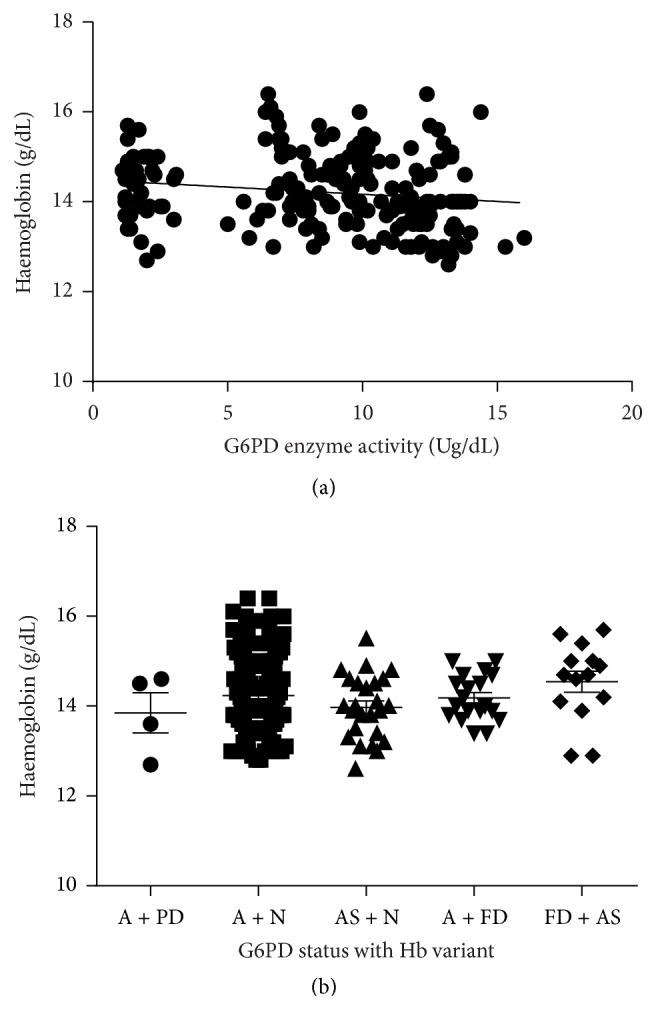
G6PD enzyme activity levels in relation to participant haemoglobin levels. (a) A scatter plot showing an inverse correlation between the participant haemoglobin levels and G6PD enzyme activity (Spearman correlation coefficient, *r* = −0.2023; *p* = 0.0041). (b) Comparing the haemoglobin levels of the participants with regard to the haemoglobin variant and/or G6PD enzymopathy status (A: haemoglobin A; S: haemoglobin S; ND: no qualitative G6PD activity; FD: full qualitative G6PD defect; PD: partial qualitative G6PD defect).

The study also found a weak but statistically significant inverse relationship between donor haemoglobin levels and red cell G6PD enzyme activity (Figure 3(a); *r* = −0.2023; *p* = 0.0041). However, when the data were stratified into the various haemoglobin variants, there was no significant difference in the haemoglobin levels between these groups (Figure 3(b)).

The 100% G6PD enzyme activity was calculated based on the adjusted G6PD-normal male median of the study participants as previously recommended [17] and is summarised in Table 5. As per the WHO recommendations, 39 (19.5%) of the participants with G6PD enzymopathy had mild G6PD deficiency, that is, 10–60% activity (Table 5) [11]. However, none had severe enzyme deficiency, that is, <10% enzyme activity.

Our study also revealed that, in our donor population, having a sickle cell trait was significantly associated with higher chance of having full qualitative red cell G6PD defect (*p* = 0.002, OR: 3.627, CI (1.630–8.067)) and mild red cell G6PD enzyme activity level (*p* = 0.038, OR: 2.410, CI (1.049–5.534)). Additionally, there was a significantly higher chance of commercial (*p* = 0.020, OR: 5.609, CI (1.309–24.035)) or voluntary donors (*p* = 0.034, OR: 2.404, CI (1.071–5.397)) having the sickle cell trait (Table 6; see also Supplementary Tables S1 and S2 in Supplementary Material available online at http://dx.doi.org/10.1155/2016/7302912).

## 4. Discussion

In the blood donor preselection protocol, prospective donors are screened using haemoglobin levels and a battery of serological tests that focusses mainly on transfusion-transmitted diseases. However, this study argues a case for the widening of the predonation screening protocol to include screening for other red cell pathologies in populations in which inheritance of such pathologies is inherently high. In this study, we show that coinheritance of sickle cell haemoglobin variant and red cell G6PD enzymopathy could be as high as 7%. Moreover, we found that 19.5% of the donor blood units are prone to stressor-induced haemolysis in any prospective recipient as a consequence of their inherent G6PD enzymopathy.

Previous studies have estimated prevalence rates of sickle cell trait and/or G6PD status in various populations. For example, a study by Omisakin et al. and Jeremiah estimated prevalence of HbS trait of 26.1 and 19.68%, respectively [14, 18], in blood donors. Egesie et al. also found SCT prevalence of 20.8% in a study that involved males in Jos in Nigeria [13]. Others have also reported SCT prevalence ranging from 20 to 40% for Ghanaian populace [12, 19]. The 19.5% prevalence rate found in our blood donors is consistent with these previous works. However, a similar work done in Ghana found a comparatively lower SCT prevalence of 11.3% in a group of 150 blood donors [20]. Also, in this study, only 3% of the participants had knowledge of their sickle cell status and haemoglobin phenotype, which agrees with a study by Lippi et al., who stated that most blood donors, especially those with SCT, were not aware of their sickle cell status [21].

With regard to G6PD enzymopathy, the WHO estimates a 15–26% prevalence rate in Ghana [11]. The 19.5% prevalence of G6PD deficiency recorded in this study confirms the prevalence rate suggested by the WHO [11] for the Ghanaian populace. Moreover, prevalence of G6PD deficiency in this study was comparable to estimated G6PD enzymopathy prevalence rates in blood donors in Osogbo, Osun State, Nigeria [22], and in Yasuj, Iran [23], that reported G6PD deficiency prevalence rates of 19.5% and 14.17%, respectively. Our prevalence rate also falls within the population-based G6PD deficiency predicted by Howes et al., for Nigeria (2%–31%), Sudan (1%–29%), and Democratic Republic of Congo (4%–32%) [10]. However, a previous cross-sectional study in Nigeria reported a relatively higher prevalence rate of 25.5% in blood donors [24]. The variance may be due to the different sample sizes (200 in the present study versus 314 in that study) and/or demographics of the studied population. In spite of the fact that G6PD enzymopathy is sex-linked and has a higher frequency in males, 93% of the blood donors in the present study were males (compared to 7% females). This is interesting as in areas with high prevalence of sex-linked enzymopathies one would expect a higher proportion of female donors to reduce the likelihood of transfusing blood with red cell enzyme deficiencies. This agrees favourably with previous studies that were undertaken in Ghana [20] and Riyadh, Saudi Arabia [25], which, respectively, found 92% and 98.7% of blood donors being males. Others have suggested such reasons as pregnancy, low body weight, and/or potential low iron levels due to menstrual cycle for the lower proportion of donors being females [26].

Presently, haemoglobin levels ≥12 g/dL are used as the threshold for predonation screening [4]. While this is necessary in ensuring that prospective donors are protected, it does not give any indication of inherited red cell haemoglobinopathies or enzymopathies. Our data shows that there is no correlation between the donor haemoglobin levels and G6PD enzyme activity or sickle cell haemoglobin trait (HbS) inheritance as well as no significant differences in the haemoglobin levels of donors with HbS trait and G6PD enzymopathy. It is estimated that most of the transfusions are used to correct malaria-induced severe anaemia [1]. Most of these cases are managed with such drugs as primaquine that can induce acute red cell haemolysis in G6PD deficient individuals with its consequent detrimental outcomes. Our study therefore strongly argues for the need to include screening for haemoglobinopathies and sickle cell trait in the predonation selection protocol in areas where genetic pressure due to malaria endemicity has led to selection of inheritance of SCT and/or G6PD enzymopathy as these offer survival advantages [5]. This is particularly important considering that prevalence of coinheritance of G6PD enzymopathy and sickle cell haemoglobin variant is estimated to be 7% in participants who donated blood at our study centre. This agrees with a previous study by Egesie et al., in Jos, Nigeria, that also found a prevalence of coinheritance of both G6PD enzymopathy and HbS variant to be 5.4% [27]. The differences in the prevalence rates could be due to the different sample sizes in the two studies; 130 blood donors in the study by Egesie et al. compared to 200 blood donors in the present study. Additionally, whereas Egesie et al. recruited donors aged between 20 and 49 years, this study recruited donors aged between 18 and 59 years. This could have also accounted for our slightly higher G6PD enzymopathy as 50.5% of our study participants were in the 18–24 years' category.

Moreover, we also found 19.5% of the donors had mild enzyme deficiency (10–60% enzyme activity). Individuals with this enzyme activity are known to undergo haemolysis when subjected to stressors like infection and/or drug therapy [11, 17]. As most of the recipients of blood in our study population may usually be suffering from severe malaria and on drug therapy, this calls for some urgency in the inclusion of G6PD and/or HbS status in all prospective donors so as to protect the potential recipients.

Our study has some limitations which include our inability to screen our G6PD deficient donors for their G6PD genotypes as these are known to impact the G6PD enzyme activity levels. Also, we acknowledge that, compared to the fluorescence spot test, the methaemoglobin reduction assay has low sensitivity with regard to heterozygous females who may have relatively high G6PD enzyme activity. Additionally, we did not make an estimate for reticulocyte counts in our study participants, although reticulocytes having remnants of RNA have higher G6PD enzyme activity. This potential confounding effect of reticulocytosis was not accounted for in our study.

## 5. Conclusion

The high prevalence of SCT and G6PD enzymopathy coinheritance demonstrated in this study suggests that screening for these inherited conditions must be incorporated into existing protocols. Individuals with rare blood groups, who might necessarily donate in spite of their haemoglobin variant and/or G6PD enzymopathy, must have their donated units appropriately labelled to minimise complications in high-risk recipients.

## Supplementary Material

S1: Logistic regression of factors associated with G6PD deficiency.
S2: Logistic regression of factors associated with co-inheritance of sickle haemoglobin variant (AS) and G6PD deficiency.

## Figures and Tables

**Table 1:** Age and gender distribution of blood donors.

Hb variant + G6PD status	Male	Female	Total

A + PD	—	4	4 (2%)
AS only	22	3	25 (12.5%)
AS + FD	14	—	14 (7%)
A + FD	19	2	21 (10.5%)
A + N	133	3	136 (68%)

Total	188 (94%)	12 (6%)	200

A and S represent haemoglobin A and haemoglobin S, respectively; N: no qualitative red cell G6PD enzyme defect; PD: partial qualitative red cell G6PD enzyme defect; FD: full qualitative red cell G6PD enzyme defect.

**Table 2:** Knowledge of blood donors on G6PD deficiency and/HbS status.

	Status		Previous donation		Total
	Yes	No	Yes	No	
G6PD	—	200	48 (24%)	152	200
Sickling	6 (3%)	194 (97%)	72 (37%)	122 (63%)	200

**Table 3:** Types of blood donors with G6PD and haemoglobin variants distribution.

Donors	G6PD-N	G6PD-D	A	AS

Commercial (*n* = 8)	3	5 (62.5%)	4	4 (50%)
Replacement (*n* = 152)	124	28 (18.4%)	128	24 (15.8%)
Voluntary (*n* = 40)	34	6 (15%)	28	12 (30%)

Total	161	39	160	40

G6PD-N: normal qualitative G6PD activity; G6PD-D: defective red cell G6PD activity; A: haemoglobin A; S: haemoglobin S.

**Table 4:** Number of donors as stratified by age (years).

Age (yrs)	Number of donors (%)

18–29	101 (50.5%)
30–39	81 (40.5%)
40–49	17 (8.5%)
50–59	1 (0.5%)

Total	200 (100%)

**Table 5:** % G6PD enzyme activity calculated from the adjusted male median of study participants.

% G6PD activity	A + PD (*n* = 4)	A + FD (*n* = 21)	AS + FD (*n* = 14)	A + N (*n* = 136)	AS + N (*n* = 25)

Median (range)	29.41 (19.61–30.39)	15.69 (10.78–25.49)	16.67 (10.78–23.53)	97.06 (49.02–156.9)	108.8 (56.86–137.3)
Mean (95% CI)	27.21 (19.11–35.3)	16.53 (14.63–18.42)	17.23 (14.43–20.02)	99.79 (95.77–103.8)	105.4 (95.82–114.9)

A and S represent haemoglobin A and haemoglobin S, respectively; N: no qualitative red cell G6PD enzyme defect; PD: partial qualitative red cell G6PD enzyme defect; FD: full qualitative red cell G6PD enzyme defect.

**Table 6:** Logistic regression of factors associated with sickle haemoglobin variant (AS).

Parameters	OR (95% CI)	*P* value

*Age group*		
18–29	0.229 (0.014–3.826)	0.305
30–39	0.250 (0.015–4.217)	0.336
40–49	0.231 (0.011–4.838)	0.345
50–59	Reference	
*Sex*		
Male	0.711 (0.183–2.758)	0.621
Female	Reference	
*G6PD status*		
Normal	Reference	
Partial defect	5.029 (5.029)	
Full defect	3.627 (1.630–8.067)	**0.002**
*G6PD activity*		
Mild deficiency	2.410 (1.049–5.534)	**0.038**
Nondeficient	0.676 (0.273–1.676)	0.398
Increased activity	Reference	—
*Donor type*		
Commercial	5.609 (1.309–24.035)	**0.020**
Voluntary	2.404 (1.071–5.397)	**0.034**
Replacement	Reference	—

OR: odds ratio; CI: confidence interval.
