# Supplementary material for: Glucose-6-Phosphate Dehydrogenase Deficiency and Sickle Cell Trait among Prospective Blood Donors: A Cross-Sectional Study in Berekum, Ghana
Source: Adv Hematol. 2016 Sep 14;2016:7302912. doi: 10.1155/2016/7302912 (PMC5039272; doi:10.1155/2016/7302912)
Supplement: Supplementary file 1 — S1: Logistic regression of factors associated with G6PD deficiency. S2: Logistic regression of factors associated with co-inheritance of sickle haemoglobin variant (AS) and G6PD deficiency. [file 7302912.f1.pdf]

Supplementary (S) data

S 1: Logistic regression of factors associated with G6PD deficiency

Parameters	OR (95% CI)	P-value
**Sex**		
Male	Reference	-
Female	4.697 (1.426-15.476)	**0.011**
**Sickling status**		
A	Reference	
AS	3.046 (1.395-6.652)	**0.005**
**Donor type**		
Commercial	7.381 (1.665-32.715)	**0.009**
Voluntary	0.782 (0.299-2.041)	0.615
Replacement	Reference	-

A: haemoglobin A; S: haemoglobin S; OR: odds ratio; CI: confidence interval

S 2: Logistic regression of factors associated with co-inheritance of sickle haemoglobin variant (AS) and G6PD deficiency

Parameters	OR (95% CI)	P-value
**Age group**		
18-29	Reference	-
30-39	1.778 (0.591-5.350)	0.306
40-49	0.000 (0.000)	0.997
50-59	-	-
**Sex**		
Male	Reference	-
Female	0.000 (0.000)	-
**Sickling status**		
Positive	1.796 (1.796)	-
Negative	Reference	-
**G6PD Activity**		
Mild deficiency	5.046 (0.000)	0.998
Non-deficient	1.000 (1.000)	-
Increased activity	Reference	-
**Donor type**		
Commercial	8.520 (1.775-40.888)	**0.007**
Voluntary	0.364 (0.045-2.932)	0.342
Replacement	Reference	-

OR: odds ratio; CI: confidence interval
